# 14-3-3 Proteins in Brain Development: Neurogenesis, Neuronal Migration and Neuromorphogenesis

**DOI:** 10.3389/fnmol.2017.00318

**Published:** 2017-10-12

**Authors:** Brett Cornell, Kazuhito Toyo-oka

**Affiliations:** Department of Neurobiology and Anatomy, Drexel University College of Medicine, Philadelphia, PA, United States

**Keywords:** 14-3-3 proteins, neurogenesis, neuronal migration, neuromorphogenesis, synaptogenesis, neurite initiation, neurite growth, neurodevelopmental disorders

## Abstract

The 14-3-3 proteins are a family of highly conserved, multifunctional proteins that are highly expressed in the brain during development. Cumulatively, the seven 14-3-3 isoforms make up approximately 1% of total soluble brain protein. Over the last decade, evidence has accumulated implicating the importance of the 14-3-3 protein family in the development of the nervous system, in particular cortical development, and have more recently been recognized as key regulators in a number of neurodevelopmental processes. In this review we will discuss the known roles of each 14-3-3 isoform in the development of the cortex, their relation to human neurodevelopmental disorders, as well as the challenges and questions that are left to be answered. In particular, we focus on the 14-3-3 isoforms and their involvement in the three key stages of cortical development; neurogenesis and differentiation, neuronal migration and neuromorphogenesis and synaptogenesis.

## Introduction

The 14-3-3 protein family consists of seven isoforms in mammals, encoded by seven separate genes, each denoted by a Greek letter (β, γ, ε, ζ, η, τ and σ). This family was given their name when these proteins were originally discovered in 1967 as abundant proteins in the mammalian brain. This name was chosen due to the particular elution and migration pattern of these proteins in DEAE-cellulose chromatography and gel electrophoresis, with the 14-3-3 proteins eluting in the 14th fraction of bovine brain homogenate on DEAE-cellulose and in position 3.3 in the gel (Moore and Perez, 1967; Aitken, 2006). For a detailed review of the history of the 14-3-3 family, an excellent review was written by Aitken (2006).

### 14-3-3 Structure

**Figure 1:**
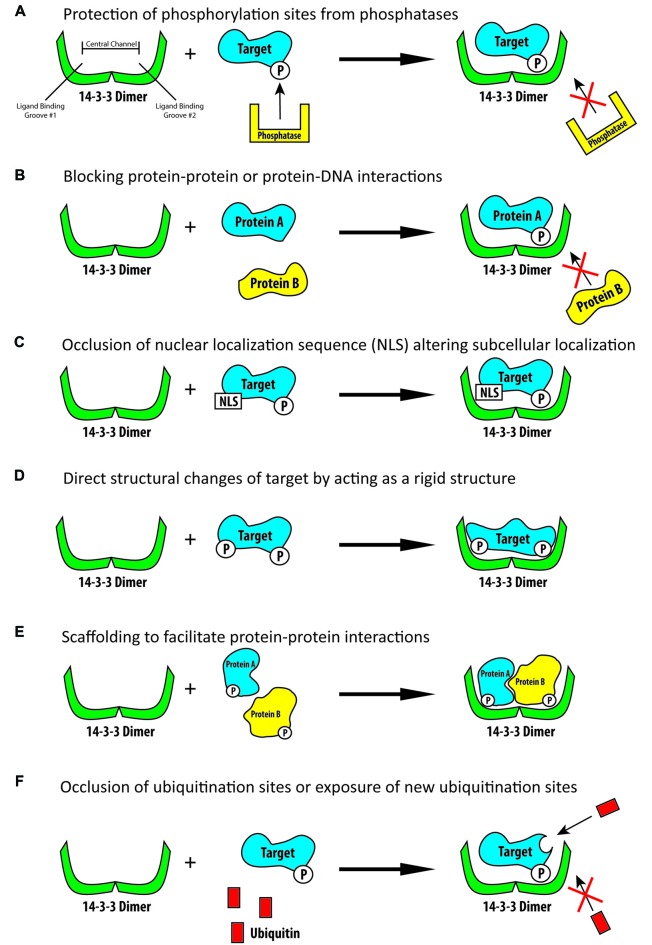
Schematic illustration of some of the known functions of 14-3-3 proteins. **(A)** 14-3-3 proteins are able to bind phosphorylated targets and prevent their dephosphorylation by phosphatases. **(B)** 14-3-3 proteins can bind phosphorylated targets and block protein-protein or protein-DNA interaction sites. **(C)** 14-3-3 proteins are able to bind their targets and block localization signals, including nuclear localization signals, thus altering their targets subcellular localization. **(D)** 14-3-3 proteins can produce direct conformational changes on their targets by acting as rigid structures. **(E)** 14-3-3 proteins can act as rigid scaffolding structures and bind multiple targets to bring them into close proximity to one another. **(F)** 14-3-3 proteins can bind their target and block ubiquitination sites thus preventing the subsequent degradation of their target or they may bind their target and increase ubiquitination and subsequent degradation.

14-3-3 proteins are acidic proteins that form and function as both homodimers and heterodimers, with the exception of the 14-3-3σ isoform, which preferentially forms homodimers (Benzinger et al., 2005; Bridges and Moorhead, 2005; Wilker et al., 2005; Gardino et al., 2006; Obsilova et al., 2008). 14-3-3 proteins are highly helical and as dimers form a characteristic cup-like structure with a large negatively charged central channel approximately 35Å in diameter and 20Å deep, containing two ligand binding grooves (Liu et al., 1995; Xiao et al., 1995; Yang et al., 2006; Obsil and Obsilova, 2011; Figure 1A). The dimeric structures of the seven 14-3-3 isoforms in mammals are very similar, however various dimers show differences in the relative position of the monomers and altered angles between the monomers thus altering their binding specificity. Furthermore, the 14-3-3 channel is formed with invariant residues between isoforms forming a consistent cup-like structure. However, the residues forming the outer surface of 14-3-3 proteins vary between isoforms and may also play a role in isoform specific interactions with target proteins (Benzinger et al., 2005; Gardino et al., 2006; Yang et al., 2006; Aitken, 2011). In addition, the N-terminal of all 14-3-3 isoforms are also highly variable and because this terminal is essential for dimer formation, this can limit the number of heterodimer combinations that can occur, with specific dimers having specific binding targets, thus conferring further specificity (Chaudhri et al., 2003; Liang et al., 2008; Fischer et al., 2009; Kligys et al., 2009; Aitken, 2011). The cup-like structure of 14-3-3 dimers is key to their function and binding of protein targets. 14-3-3 proteins typically bind phosphorylated serine and threonine consensus binding motifs on their targets with two optimal binding motifs, RSXpSXP and RX(Y/F)XpSXP (Yaffe et al., 1997; Rittinger et al., 1999). While there are two optimal consensus binding motifs, 14-3-3 proteins are also known to bind non-standard phosphorylated serine and threonine sites that do not conform to the optimal consensus motifs and in some cases can bind to non-phosphorylated targets (Fu et al., 1993; Muslin et al., 1996; Campbell et al., 1997; Masters et al., 1999). For example, 14-3-3 proteins are known to bind the protein Bax, which is a key regulator in apoptosis, in a non-phosphorylation dependent manner (Nomura et al., 2015). 14-3-3 proteins are also known to be able to bind two consensus sequences on a single target protein (Kostelecky et al., 2009; Johnson et al., 2010; Obsil and Obsilova, 2011). As a result of 14-3-3 dimerization and the numerous ways in which targets can be specified, 14-3-3 proteins are known to have over 200 binding partners, and this number is continuously increasing (Aitken, 2011). In addition to the basic 14-3-3 dimer structure, 14-3-3 proteins may also undergo post-translational modifications, such as; phosphorylation, fatty acylation and acetylation, polyglycylation and alterations following oxidative stress (Martin et al., 1993; Hamaguchi et al., 2003; Dikiy et al., 2007; Lalle et al., 2010; Musiani et al., 2011). These post-translational modifications can have a number of effects on 14-3-3 proteins, including; functional regulation, target interactions, specificity of dimerization and cellular localization (Aitken, 2011). For a more detailed and through examination of 14-3-3 protein structure, there are a number of excellent reviews covering the subject (Aitken, 2002, 2011; Obsil and Obsilova, 2011).

### Diversity of the 14-3-3 Proteins Molecular and Cellular Functions

In addition to the diversity and number of 14-3-3 binding targets, 14-3-3 proteins can facilitate a number of different functions once they have bound their targets (Pozuelo Rubio et al., 2004). Phosphorylation of a protein is one of the most prevalent posttranslational modifications of a protein and can have numerous effects on protein activity, localization and other functions. One known regulatory role of 14-3-3 proteins is their ability to physically cover phosphorylated sites on their targets, thus blocking the action of phosphatases and preventing the de-phosphorylation of the 14-3-3 targets (Figure 1A; Tzivion and Avruch, 2002; Toyo-Oka et al., 2003). When 14-3-3 proteins bind their targets they are also known to be able to physically block sequence specific features or structural features. For example, they may mask protein-protein interaction sites or protein-DNA interaction sites (Figure 1B; Obsil and Obsilova, 2011). 14-3-3 proteins can also change the subcellular localization of their binding partners (Figure 1C; Davezac et al., 2000; Brunet et al., 2002; Obsil et al., 2003). A famous example of 14-3-3 proteins ability to alter subcellular localization is their negative regulation of cdc25C, a protein important for cell cycle regulation. It was found that 14-3-3 proteins can bind cdc25C and sequester it to the cytosol, thus disrupting its function and altering cell cycle progression (Dalal et al., 1999; Muslin and Xing, 2000; Telles et al., 2009). 14-3-3 proteins are also able to change the conformation of their target proteins by acting as molecular anvils (Figure 1D). The 14-3-3 proteins themselves remain very rigid, thus acting as an anvil and allowing their targets to use the rigid 14-3-3 proteins as a base on which the target can be reshaped (Ganguly et al., 2001; Obsil et al., 2001; Obsil and Obsilova, 2011). In a similar regard, 14-3-3 proteins can act as a scaffold to anchor target proteins closely to one another and assist in creating protein complexes (Figure 1E; Ottmann et al., 2007). One way in which they are able to achieve this is by having one 14-3-3 monomer in the dimer bind one protein while the other monomer binds another protein, linking them together (Vincenz and Dixit, 1996; Fu et al., 2000; Ferl et al., 2002; Agarwal-Mawal et al., 2003). 14-3-3 proteins are also known to be able to regulate the ubiquitination and thus subsequent degradation of their targets (Figure 1F). In binding their targets 14-3-3 proteins have been shown to either increase or decrease the ubiquitination of their targets, presumably by either blocking the ubiquitination sites or the de-ubiquitination of their targets or assisting in conformational changes so as to expose additional ubiquitination sites (Mizuno et al., 2007; Foote and Zhou, 2012; Dar et al., 2014; Toyo-Oka et al., 2014; Cornell et al., 2016b). Although 14-3-3 proteins have been shown to have a great number molecular functions, to our knowledge 14-3-3 proteins are not known to have any intrinsic enzymatic properties.

The first function of 14-3-3 proteins was described in 1987 when it was found that 14-3-3 proteins can activate tyrosine and tryptophan hydroxylases, which are the rate limiting enzymes in the synthesis of dopamine and other neurotransmitters (Ichimura et al., 1987; Aitken, 2006). Quickly following this finding, it was then found that 14-3-3 proteins are also important inhibitors of protein kinase C (PKC; Aitken et al., 1990; Toker et al., 1990). Following these discoveries it has now been found that 14-3-3 proteins are essential regulators in a number of cellular pathways, including; cell cycle progression, apoptosis, metabolism control, transcriptional regulation of gene expression, DNA damage response and neural development (van Hemert et al., 2001; Comparot et al., 2003; Hermeking and Benzinger, 2006; Freeman and Morrison, 2011; Gardino and Yaffe, 2011).

14-3-3 proteins are highly conserved and share a large amount of structural similarity between isoforms, suggesting functional redundancy. However, while there is evidence showing some functional overlap, the 14-3-3 proteins in general show a surprising amount of binding target specificity and functional specificity between the isoforms. It has been found that 14-3-3 isoforms often have specific binding partners that are not able to be bound by other 14-3-3 dimer combinations (Comparot et al., 2003; Muslin and Lau, 2005; Aitken, 2006; Lau et al., 2006). For example, 14-3-3σ preferentially homodimerizes and is not able to bind and regulate the cell cycle protein cdc25C, whereas many other 14-3-3 dimers are able to bind and regulate this protein (Wilker et al., 2005). Furthermore, it has also been found that isoform specific knockouts can produce distinct phenotypes (Toyo-Oka et al., 2003, 2014; Cheah et al., 2012). For example, it has been found that 14-3-3ε and 14-3-3ζ double knockout (dKO) mice have altered fur pigmentation, including ventral gray patches, whereas to date this has not been reported in any other 14-3-3 knockout mice (Cornell and Toyo-Oka, 2016).

### 14-3-3 Proteins in Disease

With 14-3-3 proteins having so many diverse cellular and molecular roles and functions it comes with no surprise that this family is also associated with a number of human disorders. 14-3-3 proteins have been implicated in everything from cardiomyopathy and cancer to even hair pigmentation (Morrison, 2009; Kosaka et al., 2012; Cornell and Toyo-Oka, 2016). 14-3-3 proteins have also been shown to be involved in a number of neurological disorders, including; Parkinson’s disease, Alzheimer’s disease, Creutzfeldt-Jakob disease, schizophrenia and bipolar disorder (Berg et al., 2003; Foote and Zhou, 2012). The involvement of 14-3-3 proteins in a number of neurological disorders is of no surprise given the fact that 14-3-3 proteins are highly expressed in the brain, making up about 1% of the total soluble brain protein (Berg et al., 2003).

Interestingly over the last decade, evidence has now accumulated implicating the importance of the 14-3-3 family in the development of the nervous system, in particular cortical development, and have more recently been recognized as key regulators in a number of neurodevelopmental processes. In this review we will discuss the known roles of each 14-3-3 isoform in the development of the cortex, as well as the challenges and questions that are left to be answered. In particular, we will focus on the 14-3-3 isoforms and their involvement in the three key stages of cortical development; neurogenesis and differentiation, neuronal migration and neuromorphogenesis and synaptogenesis.

## Neurogenesis and Neurodifferentiation

**Figure 2:**
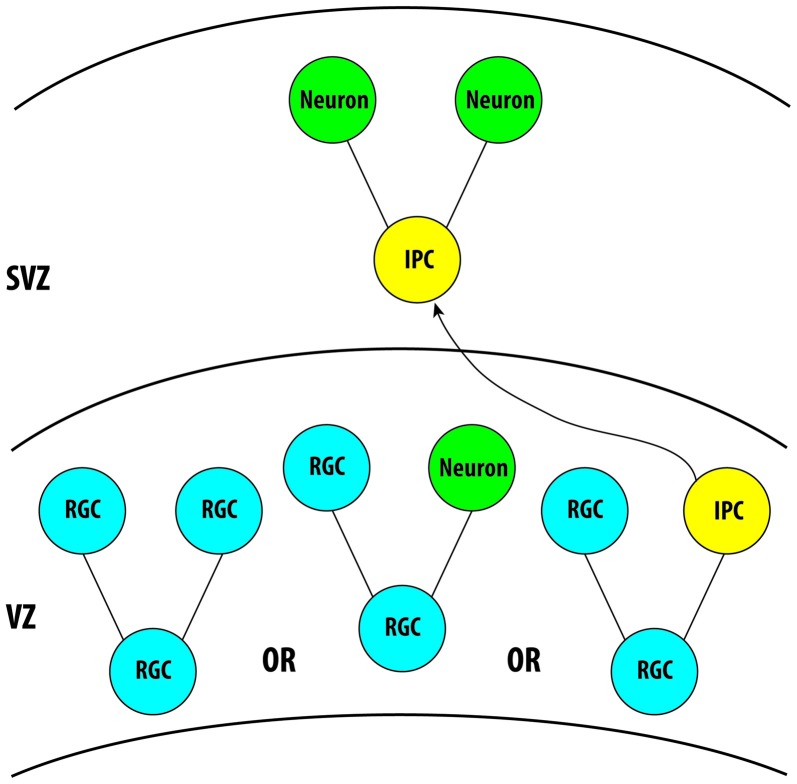
Illustration demonstrating the role of radial glial cells and intermediate progenitor cells (IPCs) in neurogenesis during cortical development. Three isoforms, 14-3-3ε, ζ and γ, are known to be expressed in these cells during cortical development. Further analysis is required for the remaining isoforms. VZ, Ventricular Zone; SVZ, Subventricular Zone.

During brain development, the majority of projections neurons that will eventually form the cortical layers of the brain are generated from neural progenitor cells localized in the ventricular zone (VZ; Kriegstein and Alvarez-Buylla, 2009). In the VZ, the division of radial glial cells (RGCs) is responsible for the generation of nearly all neurons and glial cells in the brain, either directly or indirectly (Paridaen and Huttner, 2014). During neurogenesis, RGCs will divide symmetrically to produce two RGCs, or asymmetrically to generate an RGC and a neuron either directly or indirectly through the production of an intermediate progenitor cell (IPC; Pontious et al., 2008). Following the generation of IPCs from RGCs, the IPCs will then migrate into the subventricular zone (SVZ) and typically undergo a single bout of division to generate two neurons (Toyo-Oka et al., 2014). Therefore, the asymmetric division of an RGC can either produce an RGC in addition to a neuron directly or it can divide to generate an RGC and an IPC, with that IPC subsequently symmetrically dividing and generating two neurons or two IPCs (Figure 2). In this way, RGCs and IPCs can regulate neurogenesis in order to generate the proper number of neurons in the cortex and the proper balance of proliferation and differentiation of these cells during development, which is essential for the formation of a functional brain. In addition to RGCs and IPCs, a new population of neural progenitor cells has more recently been identified, known as outer radial glial cells (oRGs). These cells are very prevalent in the human cortex beginning around mid-gestation and have been attributed to the expansion of the neocortex in primates (Nowakowski et al., 2016). Only more recently has a similar population of cells, also known as short neural progenitors (SNPs) been identified in rodents (Gal et al., 2006; Stancik et al., 2010). However, much is still to be elucidated regarding the role and the extent of this population of cells in cortical development. In this section we will review the recently discovered roles of 14-3-3 proteins in the regulation of neurogenesis and neurodifferentiation.

### 14-3-3ε and 14-3-3ζ

The 14-3-3ε isoform is encoded by the gene *YWHAE*, which is located on the 17th chromosome in humans. After the initial discovery and naming of 14-3-3ε, it was also rediscovered multiple times with newly identified functions, with the protein being temporarily called Mitochondrial Import Stimulation Factor (MSF) in rats (Alam et al., 1994; Aitken, 2006). This 14-3-3 isoform has been found in all mammalian species tested to date and is very well conserved with no known variants in the amino acid sequence (Aitken, 2006). The 14-3-3ζ isoform is encoded by the gene *YWHAZ*, which is located on the 8th chromosome in humans. Similar to the 14-3-3ε isoform, the 14-3-3ζ isoform was also rediscovered and has gone by the name Leonardo in *Drosophila* (Stomski et al., 1999; Aitken, 2006). 14-3-3ε and 14-3-3ζ have been implicated in a number of neurological disorders, including; schizophrenia, Alzheimer’s disease and Parkinson’s disease (Berg et al., 2003; Ikeda et al., 2008; Cheah et al., 2012). More recently it has been found that 14-3-3ε and 14-3-3ζ are essential regulators of neurogenesis and neurodifferentiation and may be involved in a number of neurodevelopmental disorders.

In recent work with the use of *in vitro* and *in vivo* techniques in mice, it has been found that the 14-3-3ε and 14-3-3ζ isoforms play an essential role in regulating neurogenesis during cortical development. With the use of 14-3-3ε and 14-3-3ζ single and dKO mice, Toyo-Oka et al. (2014) were able to reveal much about the 14-3-3ε and 14-3-3ζ isoforms in their role of regulating the division of RGCs and IPCs. In the single 14-3-3ε or 14-3-3ζ knockout embryos there was a nonsignificant tendency toward increased BrdU positive cells in the VZ, SVZ and intermediate zone (IZ) compared to WT mice, and with the dKO mice a significant increase in the number of BrdU positive cells was found in the VZ, SVZ and IZ indicating that there is an increased number of proliferating cells in these mice. Furthermore, they found a significant increase of Tbr2 positive cells (a marker of IPCs) in both the single knockouts as well as the dKOs. Together these initial experiments indicate that the 14-3-3ε and 14-3-3ζ isoforms are both essential in regulating the number of cells undergoing cell division as well as the number of IPCs present. Furthermore, this may suggest that these two 14-3-3 isoforms might function together in this role as a heterodimer. Although with just this evidence, there is the possibility that these isoforms are functioning in similar roles, but separately as homodimers.

Further experiments also suggest that this increase in proliferation and the number of IPCs in 14-3-3ε and 14-3-3ζ dKO mice results in an increase of neurons (Toyo-Oka et al., 2014). To examine this, an *in vivo* BrdU pulse labeling technique was used and while the single 14-3-3 knockouts did not result in an increase in neurons, the dKO mice showed an increase in new born neurons compared to WT mice. This indicates that the increased number of IPCs in the dKO mice predominantly differentiate into neurons rather than symmetrically dividing into IPCs that re-enter the cell cycle. In addition to IPCs generating neurons in the cortex, RGCs can also asymmetrically divide to produce one neuron and one RGC. Therefore, it was also examined if 14-3-3 deficiency can alter the ratio of asymmetrically vs. symmetrically dividing RGCs. One well documented method to examine asymmetric and symmetric division is to calculate the spindle orientation of RGCs as a marker of the division angle (Théry et al., 2007; Siller and Doe, 2009). Using this technique in WT mice at E15.5 shows that the majority of RGCs divide symmetrically with a spindle orientation of 70–80° relative to the plane of the VZ. However, in 14-3-3ε/ζ dKO mice there were more asymmetrically dividing RGCs with average spindle orientations of less than 70°, and with the single knockouts showing no significant difference than the controls. These data indicate that neural progenitors deficient in both 14-3-3ε and 14-3-3ζ are more likely to divide asymmetrically resulting in one neuron and one RGC rather than two RGCs. To further strengthen this evidence that 14-3-3 deficiency results in increased differentiation of neural progenitors into neurons, an *in vitro* assay was also performed to examine neurosphere differentiation in dKO progenitors. With this experiment they found that the neurospheres from the dKO mice differentiated into neurons more frequently than the WT neurospheres. Interestingly, it was also found that the brains of dKO mice overall were smaller than the WT mice at E18.5, but not at E15.5, despite the fact that neural progenitor cells are preferentially dividing into neurons in the dKO mice. Because there is no difference at E15.5, one possible explanation is that in the dKO mice the new born neurons are not surviving to E18.5. This is a likely explanation due to the known involvement of 14-3-3 proteins in the regulation of apoptosis (van Hemert et al., 2001; Gardino and Yaffe, 2011). However, further studies are required to determine the role of 14-3-3ε and 14-3-3ζ in neuronal apoptosis during development.

Next, the molecular mechanism responsible for this change in neurogenesis and neurodifferentiation in 14-3-3ε and 14-3-3ζ deficient mice was examined. Mackie and Aitken (2005) found that 14-3-3ζ binds to the protein δ-catenin at S1094. Furthermore, Kim et al. (2008) and Poore et al. (2010), identified two more δ-catenin phosphorylation sites that may also be 14-3-3 binding sites, S357 and T454 (Kim et al., 2008; Poore et al., 2010). To verify the 14-3-3ζ interaction and to determine if 14-3-3ε binds δ-catenin, and at which phosphorylation sites they bind, a series of pull-down assays was performed using δ-catenin point mutations where potential binding sites were mutated to either alanine or glutamic acid to mimic un-phosphorylated and phosphorylated binding sites, respectively (Toyo-Oka et al., 2014). With this experiment, it was found that both 14-3-3ζ and 14-3-3ε bind to δ-catenin at S1094 in a phosphorylation-specific manner. Next, it was revealed that in the 14-3-3 dKO mice, there is an increase in δ-catenin protein levels. In addition, when protein synthesis was inhibited *in vitro*, the 14-3-3ε and 14-3-3ζ overexpression accelerated the degradation of δ-catenin. One known function of 14-3-3 proteins is their ability to alter the ubiquitination and subsequent degradation of their binding partners (Foote and Zhou, 2012; Dar et al., 2014). Therefore, to examine this, a proteasome inhibitor was utilized, and it was demonstrated that δ-catenin turnover could be rescued. Furthermore, using a direct ubiquitination assay it was elucidated that the overexpression of 14-3-3ε and 14-3-3ζ resulted in the accelerated ubiquitination of δ-catenin. Together these data indicate that 14-3-3ε and 14-3-3ζ regulate the ubiquitination and subsequent degradation of δ-catenin and that the knockout of these 14-3-3 isoforms results in an increase in δ-catenin.

**Figure 3:**
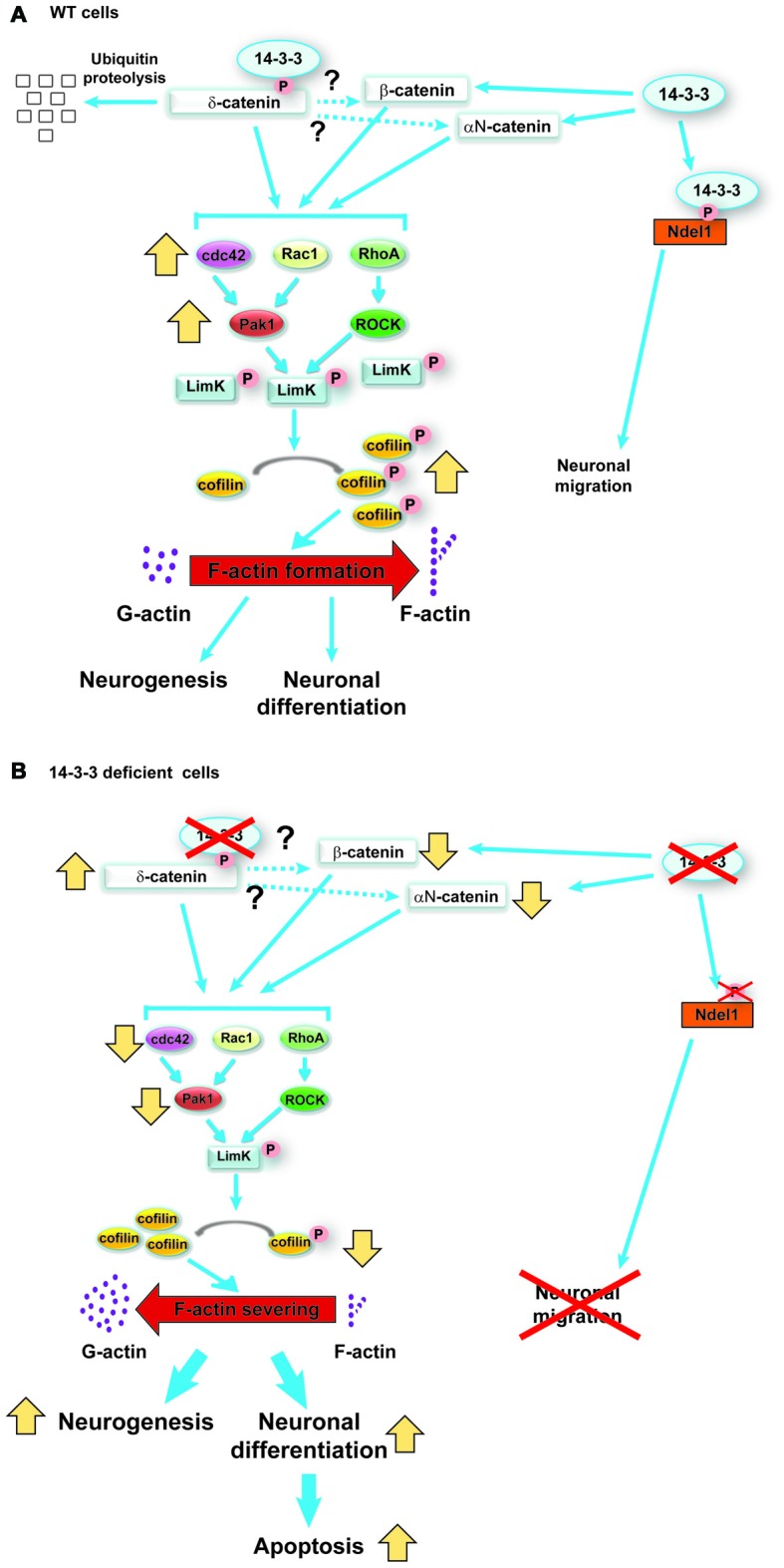
Schematic model of the functions of 14-3-3ε and 14-3-3ζ in neurogenesis and neuronal differentiation during cortical development, reproduced with permission from the Society for Neuroscience (Toyo-Oka et al., 2014). **(A)** In wild-type (WT) progenitor cells, 14-3-3ε and ζ interact with δ-catenin and regulate its ubiquitination and subsequent degradation. δ-catenin then may regulate the stability of β-catenin and αN-catenin (dotted lines). The catenin proteins then activate the Rho family of GTPases, which in turn results in the phosphorylation of Limk1 through Pak and ROCK proteins. Then, phosphorylated Limk1 phosphorylates cofilin. Phosphorylated cofilin is inactive and will not sever F-actin, resulting in accelerated F-actin formation. **(B)** In contrast, when the progenitor cells are deficient in 14-3-3ε and ζ, the δ-catenin protein levels increase. This results in an opposite cascade of events resulting in increased neurogenesis and neuronal differentiation as well as defects in the subsequent neuronal migration.

Catenin proteins are known to regulate the activity of RhoA, Rac1 and cdc42. Furthermore, the activation of Rho family GTPases results in the activation of Limk1, which once phosphorylated will then phosphorylate cofilin, inactivating it. Active cofilin (non-phosphorylated form) typically severs F-actin, therefore when cofilin is inactivated F-actin formation is accelerated (Luo, 2002). To determine if this pathway is affected by the increased levels of δ-catenin in the 14-3-3 dKO, the activity of Rho GTPases was examined, and it was clarified that in neurospheres from the dKO mice RhoA, Rac1 and cdc42 activity was decreased compared to WT. In addition the phosphorylation of Limk1and cofilin was analyzed, and it was found that their phosphorylation was decreased in the 14-3-3 dKO mice. Finally, to verify that the changes in neurogenesis and differentiation seen in the dKO mice are the result of δ-catenin altering this pathway, δ-catenin in WT neurospheres was overexpressed, and it was found an increase in differentiation of neural progenitor cells mimicking the results from the dKO mice. Additionally the knockdown of δ-catenin in neurospheres from the dKO mice normalized neuronal differentiation. The results of this study are summarized in Figure 3 (Toyo-Oka et al., 2014). Interestingly, 14-3-3 proteins, including 14-3-3ζ, have been shown to bind to Limk1 and cofilin (Gohla and Bokoch, 2002; Birkenfeld et al., 2003). Therefore, it is possible that in addition to the pathway described above through the 14-3-3 proteins interactions with δ-catenin, the 14-3-3 proteins may also be interacting with these downstream elements and further regulating this pathway and thus neurogenesis and differentiation.

Interestingly, it was shown that the 14-3-3ε and 14-3-3ζ dKO mice often displayed seizures when moved to a new cage, with the seizers typically lasting around 15 s and showing similar behaviors as a Racine Class II seizure described by Racine et al. (1979). Furthermore, EEG analysis on freely moving mice showed high frequency, high voltage rhythmic activity during the seizures in the dKO mice. These observations may indicate the involvement of 14-3-3 proteins in the onset of epilepsy.

### Other 14-3-3 Isoforms

The roles of the remaining 14-3-3 isoforms in neurogenesis and neuronal differentiation have yet to be studied and are for the most part unknown. In general, it has been well established that 14-3-3 proteins play key roles in cell cycle control, apoptosis and cancer progression in non-neuronal cells (van Hemert et al., 2001; Hermeking and Benzinger, 2006; Morrison, 2009; Freeman and Morrison, 2011; Gardino and Yaffe, 2011; Dar et al., 2014). It is reasonable to predict that 14-3-3 proteins also function in regulatory roles during neurogenesis and neuronal differentiation during cortical development. However, to date a very limited number of studies have been performed to analyze the function of 14-3-3 proteins in neurogenesis.

The 14-3-3γ isoform is encoded by the gene *YWHAG*, which is located on the 7th chromosome in humans and the protein is 100% identical to the rat ortholog (Horie et al., 1999). 14-3-3γ is highly expressed during embryonic cortical development in mice until around P7. However, by P30 14-3-3γ is minimally expressed in the cortex (Wachi et al., 2016). Interestingly, aberrant 14-3-3γ expression has been associated with a number of neurological disorders. It has been found that 14-3-3γ levels are reduced in the cortex of fetal Down Syndrome brains, however, 14-3-3γ levels have been shown to be elevated in Alzheimer’s disease (Fountoulakis et al., 1999; Peyrl et al., 2002; Berg et al., 2003). Furthermore, studies have identified 14-3-3γ as an oncogene with aberrant roles in cancer progression by indirectly down-regulating the transactivation of p53 tumor suppressor (Jin et al., 2008; Radhakrishnan and Martinez, 2010; Radhakrishnan et al., 2011). Together this evidence suggests that 14-3-3γ may play an important role in cell cycle progression and cortical development. To date, few studies have analyzed the role of 14-3-3γ in neurogenesis and neuronal differentiation during cortical development. In these studies it was found the knocking down 14-3-3γ or overexpressing 14-3-3γ *in vivo* did not produce any significant defects in cell cycle exit, proliferation and spindle orientation of neuronal progenitor cells (Cornell et al., 2016a; Wachi et al., 2016). This indicates that 14-3-3γ may not play a large regulatory role in neurogenesis, however further studies are required.

The 14-3-3σ isoform has long been known to play a regulatory role in cell cycle control. Furthermore, 14-3-3σ has been classified as a tumor suppressor and is known to be down-regulated in breast cancer (Hermeking et al., 1997; Ferguson et al., 2000; Mhawech, 2005). 14-3-3σ has been shown to bind to cdc2 complexes and sequesters cdc2 to the cytoplasm, thus enabling DNA damages to be repaired before the cell cycle progresses (Chan et al., 1999; Laronga et al., 2000; Morrison, 2009). However, 14-3-3σ is unique in the fact that it is predominantly expressed in epithelial cells and preferentially functions as a homodimer (Wilker et al., 2005). Thus it may be unlikely that the 14-3-3σ isoform is an important regulator of neurogenesis and neuronal differentiation due to its particularly low expression in the cortex.

## Neuronal Migration

Neuronal migration is responsible for the proper distribution of neurons throughout the entire nervous system and is essential for establishing the basic organization of the brain. In this section we will focus on radial migration in the cortex. Following the generation of neurons in the VZ and SVZ, these neurons must then migrate toward the cortical plate in order to form proper cortical layers. Disruptions in this process can lead to a number of disorders including; epilepsy, lissencephaly, mental retardation and brain malformations.

### 14-3-3ε and 14-3-3ζ

The importance of 14-3-3ε in neuronal migration has been fairly well established in the literature, with the first findings appearing over a decade ago in 2003 when it was found that the 14-3-3ε isoform is an essential protein for radial neuronal migration during cortical development (Toyo-Oka et al., 2003). In this work it was clarified that the deletion of the *YWHAE* gene is responsible for Miller-Dieker syndrome (MDS). Overlapping heterozygous deletions in human chromosome 17p13.3 results in two clinically distinct disorders, isolated lissencephaly sequence (ILS) and MDS. Both of these disorders result in severe neuronal migration defects which produce severe mental retardation and epilepsy in human patients. Furthermore, MDS consists of more severe symptoms than ILS, with MDS patients showing more severe cases of lissencephaly (smooth brain) as well as microcephaly and craniofacial abnormalities. Interestingly, MDS patients all have larger microscopic or submicroscopic deletions of 17p13.3 than ILS patients. A number of studies have found that the gene *PAFAH1B1* (encoding the protein *LIS1*) is essential for neuronal migration in a dose-dependent manner and is deleted in both MDS and ILS (Reiner et al., 1993; Lo Nigro et al., 1997; Hirotsune et al., 1998; Cahana et al., 2001; Gambello et al., 2003). However, as this gene is deleted in both ILS and MDS patients, this cannot explain the more severe symptoms seen in MDS patients. In 2003, it was demonstrated that the *YWHAE* gene, encoding 14-3-3ε, is deleted in all individuals with MDS, but not ILS. Furthermore, with the use of 14-3-3ε knockout mice, it was clarified that 14-3-3ε is essential for normal brain development and in particular neuronal migration. In addition it was revealed that 14-3-3ε regulates neuronal migration by binding to CDK5/p35 phosphorylated Ndel1 (also known as NUDEL) and maintaining Ndel1’s phosphorylation (Toyo-Oka et al., 2003). Ndel1 is a known LIS1 binding protein and that together they regulate cytoplasmic dynein heavy chain function, which is essential for neuronal migration (Ohshima et al., 1996; Chae et al., 1997; Niethammer et al., 2000; Sasaki et al., 2000; Gupta et al., 2002). This demonstrates that 14-3-3ε has an essential role in neuronal migration and this may explain the greater severity of symptoms seen in MDS patients.

Further experiments have verified the essential role of 14-3-3ε and perhaps 14-3-3ζ as well in neuronal migration. Using 14-3-3ε and 14-3-3ζ dKO mice, it was elucidated that these mice showed a severe disruption in cortical layering and decreased neuron travel distance from the VZ (Toyo-Oka et al., 2014). To verify that these results were the result of neuronal migration defects, a series of *in vitro* neuronal migration assays were performed, and it was demonstrated that the dKO neurons migrated much shorter distances than the WT cells. Next, because 14-3-3ε and 14-3-3ζ regulate neurogenesis and neurodifferentiation by binding to δ-catenin and preventing its degradation (see “Neurogenesis and Neurodifferentiation” section), it was tested if the defects in neuronal migration are functioning through the δ-catenin pathway, and the knockdown of δ-catenin did not rescue the migration defects. However, it was revealed that neuronal migration could be rescued by the introduction of a Ndel1 phosphomimic (see above paragraph). Together this data indicates that the neurogenesis defects found in 14-3-3 dKO mice are due to the role of these proteins binding δ-catenin, thus regulating its degradation and its downstream effect on F-actin formation through Rho family GTPases. In contrast, these 14-3-3 proteins regulate neuronal migration through a completely independent pathway by binding to the protein Ndel1.

### 14-3-3γ

The 14-3-3γ gene is highly expressed in the brain during embryonic mouse development, however its expression rapidly decreases at birth, suggesting its importance in brain development (Wachi et al., 2016). Williams Syndrome (WS) is a neurodevelopmental disorder caused by a deletion in the 7q11.23 chromosome locus resulting in developmental delay, intellectual disabilities and epilepsy. Typical WS patients (~95%) present a 1.5–1.8 Mb deletion whereas atypical patients (~5%) have a larger than 1.8 Mb deletion. Interestingly, the atypical patients with the larger deletions commonly exhibit epilepsy, and more importantly these larger atypical deletions include the gene encoding 14-3-3γ, whereas the typical deletions do not include 14-3-3γ (Fusco et al., 2014; Nicita et al., 2016). In addition to 14-3-3γ, *HIP1* has also been implicated in contributing to the more severe phenotypes in the larger atypical deletions (Ramocki et al., 2010; Fusco et al., 2014). Another interesting observation is that there is a reciprocal duplication syndrome to WS where the 7q11.23 region is duplicated, which also results in epilepsy and intellectual disabilities (Mervis et al., 1993, 2015; Morris et al., 2015). Importantly, it is well known that neuronal migration disorders are strongly correlated with the appearance of epilepsy (Palmini et al., 1991). Together these data suggest the involvement of 14-3-3γ in the epilepsy phenotype seen in the atypical WS patients.

Recent work by Cornell et al. (2016a) and Wachi et al. (2016) has now demonstrated that 14-3-3γ plays an important role in neuronal migration and that the knockdown or the overexpression of 14-3-3γ results in delays in neuronal migration *in vivo*. In these studies the researchers utilized the *in utero* electroporation (IUE) technique to examine 14-3-3γ in radial neuronal migration in developing mouse cortices. IUE is an excellent technique for transfecting and altering neuronal progenitors in the VZ prior to their migration to the cortical plate so as to visualize and study their migration. For more information on the IUE technique and its uses, Tabata and Nakajima (2001, 2008) provide excellent descriptions. Using this technique, 14-3-3γ was knocked down at E14.5 or E16.5 and then the mouse brains were harvested at P3 or P15. When scramble-shRNA was electroporated at E14.5 and the brains were harvested at P3, all of the neurons that were transfected in the VZ had successfully reached the cortical plate and formed a clear cortical layer. However, when 14-3-3γ was knocked down and analyzed at the same time points, a significant portion of the neurons was still spread throughout the IZ between the VZ and the cortical plate and had not reached the cortical plate. Furthermore, when the same experiment was performed but IUE was performed at E16.5 instead of E14.5, an even more severe phenotype was observed with almost all of the 14-3-3γ neurons still in the VZ or IZ at P3 and almost no neurons in the cortical plate, unlike the control. This indicates that 14-3-3γ may be more important in the later stages of development. Interestingly, when the same experiment was performed again but the brains were harvested at P15 instead of P3, nearly all the 14-3-3γ-deficient neurons had reached the cortical plate, matching what is seen in the controls. This indicates that the ablation of 14-3-3γ results in a delay or decreased rate of migration. To further examine this, time-lapse live imaging on brain slices following IUE was performed in order to observe neuronal migration. In this experiment IUE was performed at E14.5 and live imaging was performed at E17. It was found that neurons deficient in 14-3-3γ migrated with a significantly decreased velocity compared to control neurons. Together this data indicates that 14-3-3γ plays an important role in neuronal migration.

Interestingly, to expand upon these observations, when 14-3-3γ is knocked down; a nearly identical neuronal migration phenotype was identified when 14-3-3γ was overexpressed *in vivo* (Cornell et al., 2016a; Wachi et al., 2016). Using the same IUE time points, 14-3-3γ was overexpressed, and it was found that its overexpression produced significant delays in neuronal migration, mimicking what was seen when 14-3-3γ was knocked down. One difference being that the overexpression of 14-3-3γ did not produce more severe migration defects at later time points compared to earlier time points as seen when it was knocked down. These data indicate that a delicate balance of 14-3-3γ is required for proper neuronal migration. However, how 14-3-3γ is regulating neuronal migration and through what pathway it is acting is still unknown. Multiple 14-3-3 isoforms are known to be involved in cytoskeletal dynamics, including during neuronal migration, typically by interacting with microtubule binding proteins (Hashiguchi et al., 2000; Sluchanko and Gusev, 2010; Cornell et al., 2016b). 14-3-3γ has been found to interact with and likely bind cytoplasmic linker proteins (CLASPs; Jin et al., 2004). CLASPs are microtubule associated proteins that bind to the plus tips of microtubules and through this action can regulate the density and length of microtubules as well as promote stability (Mimori-Kiyosue et al., 2005). While the effects of 14-3-3γ binding to CLASPs is unknown, it is possible that this interaction may be altering microtubule dynamics and thus disrupting neuronal migration when 14-3-3γ levels are altered.

### Other 14-3-3 Isoforms

To our knowledge the remaining 14-3-3 isoforms (β, η, τ and σ) have not been studied in regard to their involvement in neuronal migration. However, some studies have been performed using non-neuronal models indicating that 14-3-3ε, ζ and γ may not be the only isoforms involved in regulating neuronal migration during cortical development.

The 14-3-3β isoform is encoded by the gene *YWHAB* located on the 20th chromosome in humans at the 20q13.12 locus. One of the first indications of the involvement of 14-3-3β in migration was from a few studies that found that 14-3-3β binds to the cytoplasmic tail of β1-integrin and that the overexpression of 14-3-3β can stimulate cell spreading and migration *in vitro* in non-neuronal cells (Han et al., 2001; Rodriguez and Guan, 2005). Integrins are a family of cell surface proteins that link the extracellular matrix to the actin cytoskeleton and due to this connection; integrins can regulate cell migration (Cary et al., 1999; Jacquemet et al., 2013). In addition to the interaction between 14-3-3β and β1-integrin, 14-3-3β may also be able to promote cell migration by interacting with the proteins Raf-1 or p130cas (Mhawech, 2005). Furthermore, 14-3-3β has been implicated in regulating the migration of human malignant glioma cells with the expression levels of 14-3-3β being proportional to the migratory ability of these cells (Park et al., 2012). Whether or not these mechanisms play a role in neuronal migration during cortical development has yet to be investigated.

The 14-3-3τ isoform is encoded by the gene *YWHAQ* located on the 2nd chromosome in humans at the 2p25.1 locus. 14-3-3τ has been primarily studied in regard to its roles in cancer. In particular it has been identified as being frequently overexpressed in human breast cancer and its overexpression has been linked to shorter patient survival (Wang et al., 2010). Interestingly, it has been found that when 14-3-3τ is overexpressed in breast cancer cells that express 14-3-3τ at low levels, the cells show an increase in motility and a reduction in adhesion. Furthermore, the depletion of 14-3-3τ has been shown to block breast cancer cell migration (Xiao et al., 2014). In this study, Xiao et al. (2014) found that 14-3-3τ can produce these effects on cell migration in part by binding to and inhibiting RhoGDIα, thus promoting Cdc42 activation. Together, these studies provide incentive to further examine the role of 14-3-3 proteins in neuronal migration and their possible relation to developmental disorders.

## Neuromorphogenesis and Synaptogenesis

Once neurons have completed their migration from the VZ to the cortical plate to form cortical layers, they then must grow a complex dendritic arbor and extend long axons to their targets in a process collectively known as neuromorphogenesis. Following the extension of their dendrites, these neurons then must grow dendritic spines and form synaptic connections. Together, these processes are essential for the formation of a functional and immensely complex brain.

### 14-3-3ε

17p13.3 microduplication syndrome is a newly identified genetic syndrome that is characterized by duplications of various size in the 17p13.3 chromosome locus. Patients with this syndrome exhibit severe neural developmental disorders, including autism spectrum disorder (ASD), epilepsy and mental retardation (Roos et al., 2009; Bruno et al., 2010; Avela et al., 2011; Hyon et al., 2011; Curry et al., 2013; Eriksson et al., 2015; Ibitoye et al., 2015; Przybylska-Kruszewska et al., 2016). Interestingly, Bruno et al. (2010) were able to identify a 72 Kb microduplication critical region within the 17p13.3 locus that exclusively contains the gene encoding 14-3-3ε. This strongly implicates the involvement of 14-3-3ε in 17p13.3 microduplication syndrome and ASD.

**Figure 4:**
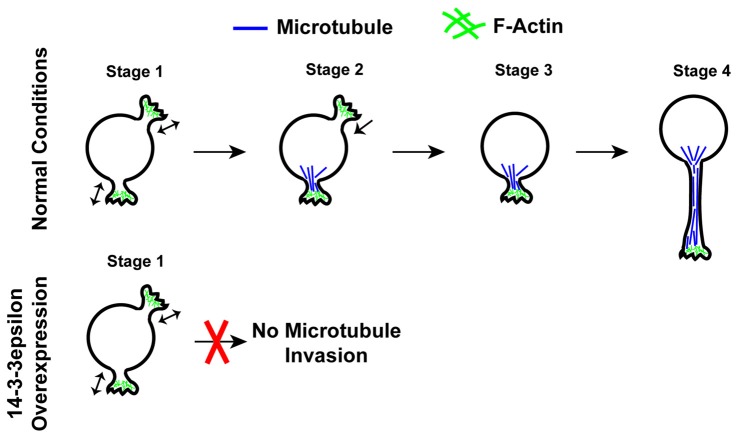
Schematic illustration of neurite initiation. Illustration of the typical stages of neurite initiation. In stage 1, actin based lamellipodia type structures rapidly form and retract. In stage 2, microtubules begin to invade and stabilize lamellipodia structures preventing their collapse. In stage 3, neurites that have been invaded by microtubules become stable structures and begin to extend in stage 4. The overexpression of 14-3-3ε prevents the invasion of microtubules into forming neurites as seen in stage 2, thus disrupting neurite formation.

**Figure 5:**
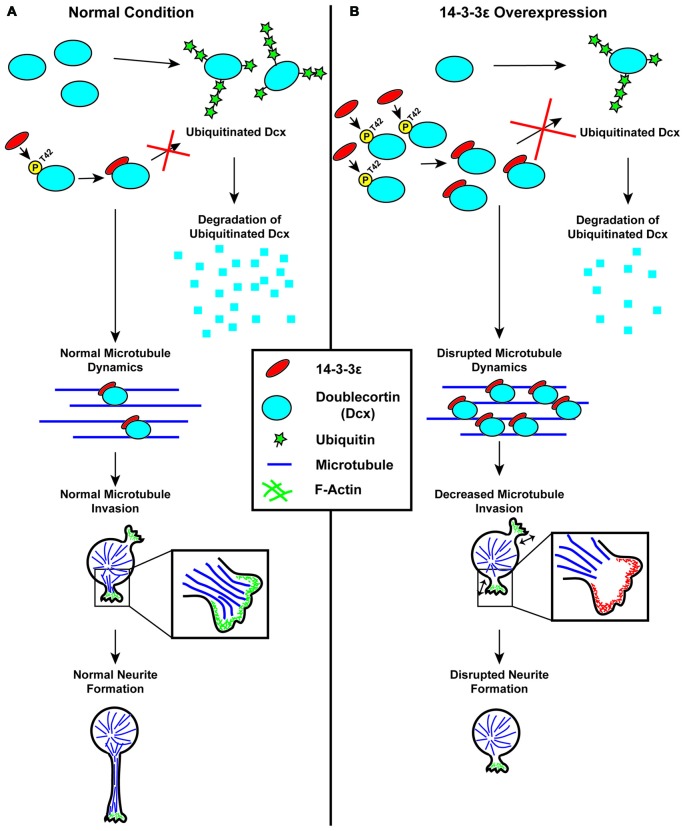
Schematic illustration of the regulation of neurite initiation by 14-3-3ε during cortical development. **(A)** Under normal conditions, 14-3-3ε binds to doublecortin (Dcx) at phosphorylated threonine-42 (P-T42). The remaining Dcx that is not bound to 14-3-3ε is rapidly ubiquitinated and subsequently degraded. Dcx stabilized by 14-3-3ε will then bind to microtubules, allowing for normal microtubule dynamics. During neurite initiation, microtubules are able to enter and stabilize lamellipodia allowing for normal neurite initiation. **(B)** When 14-3-3ε is overexpressed, there is increased binding of 14-3-3ε to Dcx, preventing its ubiquitination and subsequent degradation, resulting in an increase in Dcx protein levels. The increased Dcx binds to microtubules in excess, and this disrupts normal microtubule dynamics. This prevents microtubules from invading lamellipodia type structures during neurite initiation thus inhibiting normal neurite formation.

Recently Cornell et al. (2016b) have found that 14-3-3ε is an important regulator of neuronal morphogenesis and that the overexpression of 14-3-3ε can severely disrupt the initiation of neurite formation (Figure 4). In this work, first it was identified that the overexpression of 14-3-3ε *in vivo* in mice via IUE results in neurons in the cortical plate at P15 having significantly fewer neurites protruding from the soma and a significant decrease in mean neurite length. Furthermore, they found that the ablation of 14-3-3ε with the use of 14-3-3ε conditional knockout mice results in increased neurite length and number of primary neurites protruding from the soma. These data indicate that 14-3-3ε is an important regulator in neuronal morphogenesis. Next, time-lapse live imaging on brain slices following IUE was utilized, and it was revealed that the overexpression of 14-3-3ε severely disrupts neurite initiation, resulting in neurons undergoing continual bouts of extension and retraction of filopodia and lamellipodia type structures that fail to extend and form distinct neurites. Next, using a series of immunoprecipitation experiments using truncation and point mutants it was demonstrated that 14-3-3ε binds to the protein Doublecortin (Dcx) at T42 in a phosphorylation specific manner. Doublecortin is a known microtubule binding protein and that is overexpression can disrupt the organization and polymerization of microtubules (Gleeson et al., 1999; Horesh et al., 1999). Furthermore, it was shown that the overexpression of 14-3-3ε results in an increase in Dcx protein levels and that this is the result of 14-3-3ε binding Dcx and preventing its ubiquitination and subsequent degradation. Interestingly, the overexpression of Dcx mimicked the neuromorphological defects seen when 14-3-3ε is overexpressed. Furthermore, the knockdown of Dcx in 14-3-3ε overexpressing neurons *in vivo* rescued the number of neurites protruding from the soma; however it did not rescue the mean neurite length. Together this indicates that the overexpression of 14-3-3ε disrupts neurite initiation by binding to and preventing the degradation of Dcx, but that a separate mechanism may be responsible for the subsequent neurite extension. During neurite initiation, actin based filopodia and lamellipodia will rapidly undergo bouts of formation and retraction followed by the invasion of microtubules into these formations to stabilize them and prevent their retraction (Dehmelt and Halpain, 2004; Flynn, 2013). With the use of time-lapse live imaging *in vitro*, it was found that when 14-3-3ε is overexpressed microtubules fail to invade lamellipodia type structures during initiation. Furthermore, knocking down Dcx in 14-3-3ε overexpressing cells could rescue the invasion of microtubules into these structures. These results are summarized in Figure 5 (Cornell et al., 2016b). Together these data indicate that 14-3-3ε is an important regulator of neuronal morphogenesis and that the overexpression of 14-3-3ε disrupts neurite initiation by binding to Dcx, preventing its degradation resulting in increased levels of Dcx, thus disrupting microtubule dynamics and preventing their invasion into primitive neurites.

Interestingly, Pramparo et al. (2011) found that the knockout of 14-3-3ε in mice resulted in an increase in synaptogenesis. In this study, Pramparo et al. (2011) immunostained the cortices of the 14-3-3ε knockout mice with the synaptic markers α-synuclein and synaptophysin. They found that the 14-3-3ε knockout mice show much stronger staining than the WT mice, indicating an up-regulation of synaptogenesis. This is consistent with the findings of Cornell et al. (2016b), as they found that the knockout of 14-3-3ε results in an increase in neurite length and an increase in the number of neurites. These morphological changes may amplify the results seen by Pramparo et al. (2011) as the increased number and length of dendrites provides a greater surface area for synaptogenesis. Furthermore, it is possible that the up-regulation of synaptogenesis identified by Pramparo et al. (2011) may be the result of a similar mechanism as described by Cornell et al. (2016b) in neurite initiation, with dendritic spine formation rather than neurite initiation. However, further research is needed to determine the mechanisms underlying this upregulation of synaptogenesis. It is important to note, however, that alterations in dendritic spine density and synapse formation is often associated with ASD.

### 14-3-3ζ

A number of studies have shown an association between the 14-3-3ζ isoform and the development of schizophrenia, with 14-3-3ζ knockout mice becoming a model for schizophrenia (Jia et al., 2004; Middleton et al., 2005; Cheah et al., 2012; Fromer et al., 2014). More recently it has also been found that knocking out 14-3-3ζ in mice results in brain morphological changes, including a decrease in hippocampal dendritic spine density (Jaehne et al., 2015; Xu et al., 2015). In 2015, using 14-3-3ζ knockout mice, Jaehne et al. (2015) found that these mice have a significant decrease in dendritic spine density in the cornu ammonis layer 3 (CA3) of the hippocampus (Jaehne et al., 2015). Furthermore, Xu et al. (2015) generated a 14-3-3ζ knockout mouse model with a BALB/c background and found that these mice have enlarged lateral ventricles, aberrant mossy fiber connectivity and reduced synaptic density in all of the subfields of the hippocampus (Xu et al., 2015). These morphological symptoms are all hallmarks of schizophrenia, further validating the usefulness of this mouse model in studying schizophrenia, and also providing further evidence of the involvement of 14-3-3ζ in the formation of dendritic spines.

### Other 14-3-3 Isoforms

Very little is known regarding the involvement of the remaining 14-3-3 isoforms in neurite and synapse formation. Prior to the studies described above regarding 14-3-3ε in neuromorphogenesis, Yoon et al. (2012) found that the competitive inhibition of all 14-3-3 isoforms using a peptide inhibitor (R18) in retinal ganglion cells impairs axon elongation *in vitro* and *in vivo*. However, what specific 14-3-3 isoforms are responsible for this effect are unknown. In addition, the ablation of 14-3-3γ in cortical neurons *in vivo* resulted in abnormal neuronal morphology with shorter leading processes and fewer processes reaching the marginal zone (Wachi et al., 2016). Qiao et al. (2014) generated a 14-3-3 functional knockout mouse model in which a 14-3-3 isoform-independent inhibitor peptide is expressed in the brain, functionally inhibiting all 14-3-3 proteins. While studying these mice, Foote et al. (2015) found that not only do these mice display behavioral deficits corresponding to schizophrenia, but also show a reduction of dendritic spine complexity and spine density in forebrain excitatory neurons. This study further demonstrates the importance of 14-3-3 proteins in neuromorphogenesis. In contrast to these studies where 14-3-3 proteins were ablated or inhibited, the overexpression of any individual 14-3-3 isoform, other than 14-3-3ε, in cortical neurons *in vivo* did not produce any obvious morphological changes in regard to neurite length or the number of neurites protruding from the soma (Cornell et al., 2016b). However, the independent ablation of the individual isoforms may reveal more information as to the function of these isoforms in neuromorphogenesis. 14-3-3 proteins are known to frequently be involved in the regulation of cytoskeletal dynamics and are highly expressed in neurons during neurite formation and synaptogenesis suggesting that they may play important roles in these processes.

## Challenges and Unsolved Questions

The members of the 14-3-3 protein family perform a vast array of functions by binding hundreds of target proteins throughout the body. This family has more recently been found to play essential roles in the developing cerebral cortex and human neurodevelopmental disorders. However, due to the multitude of functions and targets, there is still much that is unknown about this protein family. In particular, a number of the 14-3-3 isoforms have yet to be analyzed in regard to their involvement in any of the three stages of cortical development; neurogenesis, neuronal migration and neuromorphogenesis (Table 1).

**Table 1:** Summary of the involvement of 14-3-3 isoforms in three stages of cortical development and each isoform potential involvement in related neurodevelopmental disorders.

	Neurogenesis	Neuronal migration	Neuromorphogenesis	Potential involvement in neuro-developmental disorders	References
14-3-3ε	+	+	+	Miller-Dieker Syndrome 17p13.3 Microduplication Syndrome Autism Spectrum Disorder Epilepsy Schizophrenia	Toyo-Oka et al. (2003, 2014), Ikeda et al. (2008) and Cornell et al. (2016b)
14-3-3ζ	+	+	N/A	Epilepsy Schizophrenia	Cheah et al. (2012) and Toyo-Oka et al. (2014)
14-3-3γ	N/A	+	N/A	Atypical Williams Syndrome 7q11.23 Duplication Syndrome Fetal Down Syndrome	Fountoulakis et al. (1999), Peyrl et al. (2002) and Cornell et al. (2016a)
14-3-3σ	N/A	N/A	N/A	N/A	
14-3-3β	N/A	N/A	N/A	N/A	
14-3-3η	N/A	N/A	N/A	N/A	
14-3-3τ	N/A	N/A	N/A	N/A	

*“+” denotes the known involvement of a particular isoform in the associated developmental stage. “N/A” indicates that the relationship is Not Determined and that further research is needed*.

One challenge that is presented when studying 14-3-3 proteins is the fact that 14-3-3 proteins function as homodimers and heterodimers. In this regard, 14-3-3 isoforms may have functional redundancy. However, 14-3-3 isoforms also show target specificity depending on the particular heterodimer or homodimer. Furthermore, alterations in the levels of a particular isoform may produce indirect effects by changing the balance of the 14-3-3 population resulting in an altered distribution of heterodimers and homodimers as non-preferential dimers are formed when the preferred dimer is no longer possible. To further confound the situation, 14-3-3 proteins also undergo post-translational modifications that can alter their function and binding specificity. In addition, 14-3-3 isoforms are known to have multiple targets and thus can act upon multiple signaling pathways simultaneously. Together, the vast functionality of these simple dimers provides a number of challenges when studying their roles in cortical development and developmental disorders. The importance of the 14-3-3 family in cortical development and the challenges involved in studying this family leaves much to be elucidated.

## Author Contributions

BC wrote the manuscript, and KT finalized it.

## Conflict of Interest Statement

The authors declare that the research was conducted in the absence of any commercial or financial relationships that could be construed as a potential conflict of interest.
